# Environmental, socioeconomic, and sociocultural drivers of monkeypox transmission in the Democratic Republic of the Congo: a One Health perspective

**DOI:** 10.1186/s40249-025-01278-9

**Published:** 2025-02-07

**Authors:** Guangyu Lu, Zeyin Chong, Enyu Xu, Ce Na, Kaixuan Liu, Liying Chai, Pengpeng Xia, Kai Yang, Guoqiang Zhu, Jinkou Zhao, Olaf Müller

**Affiliations:** 1 School of Public Health, Medical College of Yangzhou University, Yangzhou University, Yangzhou City, China; 2 Jiangsu Key Laboratory of Zoonosis, Yangzhou, China; 3 College of Information Engineering, College of Artificial Intelligence, Yangzhou University, Yangzhou, China; 4 College of Veterinary Medicine, Yangzhou University, Yangzhou, China; 5 Joint Laboratory of International Cooperation On Prevention and Control Technology of Important Animal Diseases and Zoonoses of Jiangsu Higher Education Institutions, Yangzhou University, Yangzhou, China; 6 The Global Fund to Fight AIDS, Tuberculosis and Malaria, Geneva, Switzerland; 7 Institute of Global Health, Medical School, Ruprecht-Karls-University Heidelberg, Heidelberg, Germany

**Keywords:** Human monkeypox, Mpox, One Health, Risk analysis, Grey prediction model, Democratic Republic of the Congo

## Abstract

**Background:**

Monkeypox (mpox) is an emerging zoonotic disease that has persistently impacted public health in endemic regions of West and Central Africa for over half a century. The Democratic Republic of the Congo (DRC) remains one of the countries most affected. Understanding the risk factors for disease transmission from a One Health perspective is of great importance in the risk assessment, prevention, and control of zoonotic diseases. Therefore, this study aimed to investigate the risk factors for human mpox transmission at the human–animal–environment interface in the DRC.

**Methods:**

Epidemiological, environmental, socioeconomic, and sociocultural data from the DRC from 2000 to 2015 were obtained from publicly available dataset. Using these data, we applied negative binomial regression model, least absolute shrinkage and selection operator regression model, and principal component analysis (PCA) to identify key environmental, socioeconomic, and sociocultural factors contributing to mpox transmission. Moreover, a grey prediction model GM (1, n) was constructed to predict the epidemic trend of mpox post-2015 and validated using suspected mpox case data in the DRC from 2016 to 2021, sourced from the United States Centers for Disease Control and Prevention.

**Results:**

Between 2000 and 2021, a total of 43,628 suspected mpox cases were reported in the DRC, with a peak of 6216 cases in 2020. From 2016 to 2021, suspected cases accounted for over half (24,379/43,628, 55.9%) of the total reported during the 2000–2021 period. The proportion of primary forest [incidence rate ratio (IRR): 1.023, 95% confidence interval (*CI*): 1.018–1.027], index of economic well-being (IRR: 1.046, 95% *CI*: 1.039–1.052), and mean annual precipitation (IRR 1.040, 95% *CI:* 1.031–1.049) were positively associated with mpox incidence. PCA identified five principal components, explaining 69% of the variance in the environmental, socioeconomic, and sociocultural variables. The first component was characterized by socioeconomic factors. The GM (1, n) model, based on the proportion of primary forest, index of economic well-being, and mean annual precipitation, predicted the epidemic trend (revealed relative error: 2.69).

**Conclusions:**

Both socioeconomic and environmental factors play important roles in mpox transmission. Our study further highlighted the importance of considering the interconnectedness among humans, animals, and the environment, and treating these factors as a whole to explain the transmission and emergence of mpox outbreaks in the DRC according to the One Health concept.

## Background

Human monkeypox (mpox) is a viral zoonosis caused by the monkeypox virus (MPXV). MPXV infection is typically transmitted to humans through spill-over events from animals, including rodents, squirrels, and non-human primates [1–3]. Since the first case of human mpox was reported in 1970 in the Democratic Republic of the Congo (DRC), the disease has caused sporadic infections and outbreaks, mainly limited to certain countries in West and Central Africa [1, 4]. Although the global outbreak of MPXV infection in 2022 (mpox clade IIB) occurred outside the usual endemic zones in Africa [5–9], to date, the DRC remains one of the most affected countries, together with the Central African Republic, Nigeria, and the Republic of the Congo [10]. On 11 May 2023, the World Health Organization (WHO) declared that the mpox clade IIB outbreak is no longer a public health emergency of international concern (PHEIC) [11]. However, in an important development, the WHO reinstated its PHEIC status on 14 August 2024 in response to a massive resurgence of mpox clade IB cases in the DRC and a rising incidence in several other African countries [12]. Mpox clade IB causes a much higher mortality compared with clade II, and by early August 2024, DRC had already reported already approximately 14,000 cases including around 450 deaths [13, 14].

Since the cessation of mass smallpox vaccination campaigns in 1980, the incidence of human mpox has increased dramatically in the DRC [15]. This can be explained by a significant decline in herd immunity to poxviruses after the cessation of smallpox vaccination [15–17]. Other explanations include changes in natural environmental factors in the DRC and patterns of interaction between humans and nature that favor human contact with MPXV reservoirs, which may increase the probability of transmission of the virus [18, 19]. To date, most available studies have investigated the effect of environmental factors alone on the transmission of mpox [19–21]. For example, Levine et al. [20] first used an ecological niche modeling approach and found that mean annual precipitation was the key factor in the prediction of an mpox occurrence. Later, Fuller et al. [21] used remote sensing and epidemiological surveillance data and determined that forest vegetation was the most important ecological variable for the MPXV niche. Nakazawa et al. [19] further combined geo-referenced data with physical variables to determine the spatial distribution of the disease in the DRC and found that rainforest is important for mpox transmission.

The One Health concept has been proposed as an ideal strategy for the risk assessment, prevention, and control of zoonotic diseases, such as mpox [22–24]. Researchers have used this framework to better understand risk factors associated with the transmission of mpox. For example, Quiner et al. [25] investigated the introduction of MPXV in the human living environment using proxies for lifestyle and socioeconomic factors in mpox-affected areas of rural DRC. In 2022, Mandja et al. [26] investigated the associations between physical, socioeconomic, and sociocultural risk factors and annual mpox incidence at the health zone (HZ) level in the DRC using the generalized linear mixed model (GLMM). They revealed that primary forest, index of economic well-being, and temperature were positively associated with annual mpox incidence [26]. However, as the number of candidate predictors often increases to a great degree in such analysis settings, it is challenging to efficiently explore the model space using a GLMM [27]. In this case, least absolute shrinkage and selection operator (LASSO) regression analysis shows better performance in compiling high dimensionality and multicollinearity variables [28, 29]. Moreover, principal component analysis (PCA) allows a set of correlated variables to be reduced into uncorrelated components made up of subsets of these variables [30]. In line with this, we aimed to better understand the effect of environmental, socioeconomic, and sociocultural factors on human mpox transmission in the DRC using the multiple techniques at the human–animal–environment interface. In addition, the epidemiological data, alongside environmental, socioeconomic, and sociocultural variables from 2000 to 2015 in the DRC, were entered into the multivariable grey prediction model GM (1, n) to predict the epidemic trend of mpox in the DRC from 2016 to 2023, as this model is suitable for predicting future values by analyzing dependencies and interactions with other variables [31, 32].

## Methods

### Study area

**Fig. 1:**
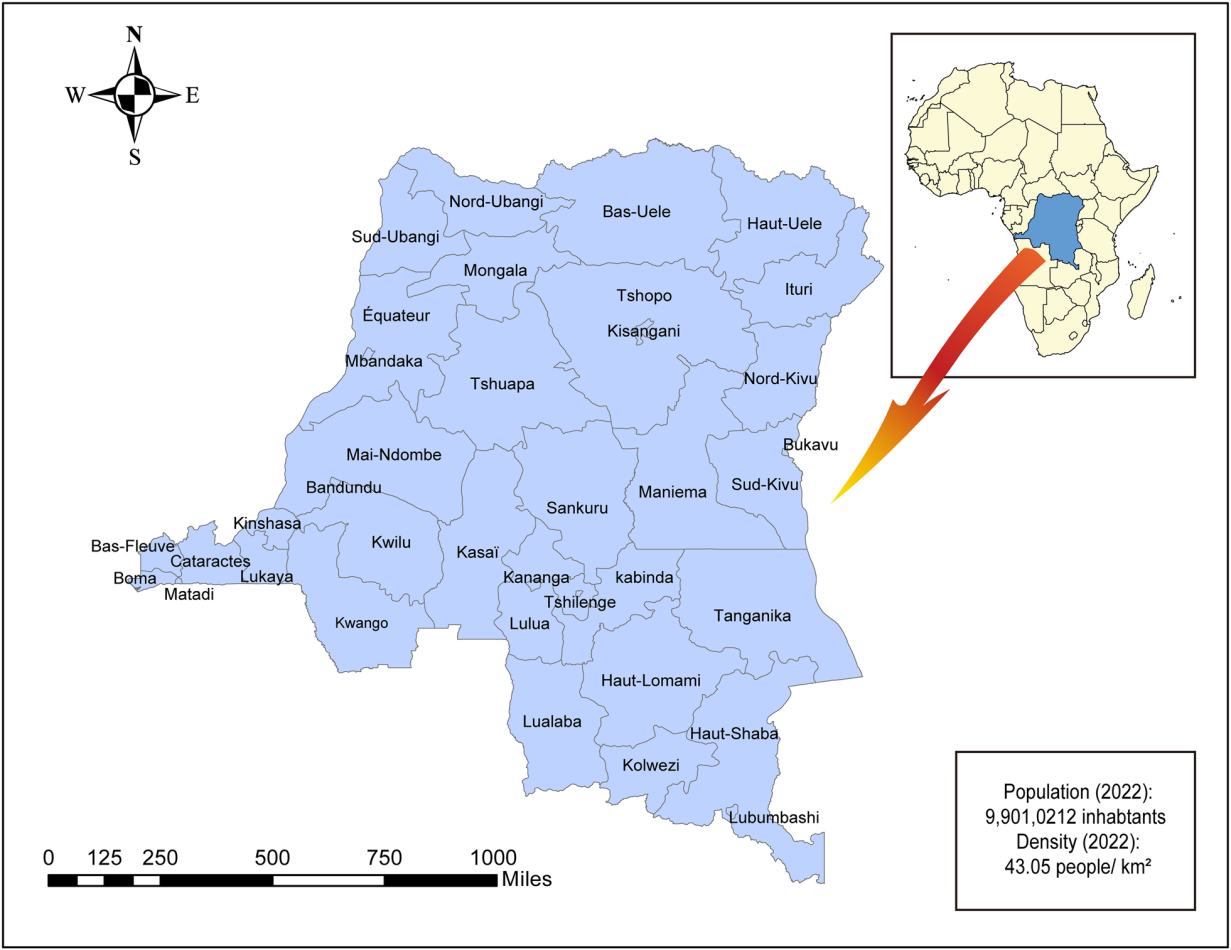
Administrative map of the Democratic Republic of the Congo with its 26 provinces. Source: The shapefile of the provinces was obtained from the free, open platform Common Geographical Reference (http://www.diva-gis.org/gdata). The map was created using the free version of ArcGIS 10.7 software (Environmental Systems Research Institute, Redlands, USA)

The DRC is the second largest country in Africa covering 2.3 million km^2^, with a population of 99,010,212 inhabitants in 2022 [33]. Most of the population in the DRC resides in rural areas with adverse economic conditions [34]. The country is divided into 26 provinces and 515 health zones (HZs), with each HZ containing 15–20 health areas (Fig. 1). The Congo Basin rainforest comprises 48% of the DRC. The vast network of rivers and other bodies of water make up the main natural environmental characteristics of the DRC. The country experiences four distinct climates. Specifically, a hot and humid equatorial climate predominates in the central basin. The north and south regions have a tropical wet climate. The east region has a temperate tropical climate. Finally, the far east region has a cool and dry highlands climate [35].

### Case definition

Suspected mpox cases include any person presenting with a high fever of sudden onset, followed a few days later by a vesicular-pustule rash that predominates on the face and is also present on the palms of the hands and soles of the feet; or the presence of at least 5 smallpox type scabs [26].

### Data sources

Our study used data from a publicly accessible dataset, obtained from the published article by Mandja et al., which encompasses epidemiological information on suspected mpox cases, along with 14 environmental, socioeconomic, and sociocultural variables at the HZ level in the DRC from January 2000 to December 2015 [26]. The weekly cases of mpox were collected by the Ministry of Public Health of DRC (through Direction of Disease Control) and were aggregated according to HZ and by year.

Data on suspected mpox cases from 2016 to 2021 were sourced from the United States Department of Health and Human Services-Centers for Disease Control and Prevention [37]. The data on suspected mpox cases from 2000 to 2021 were all from national surveillance data of the DRC and were verified with country surveillance teams [26, 37].

**Fig. 2:**
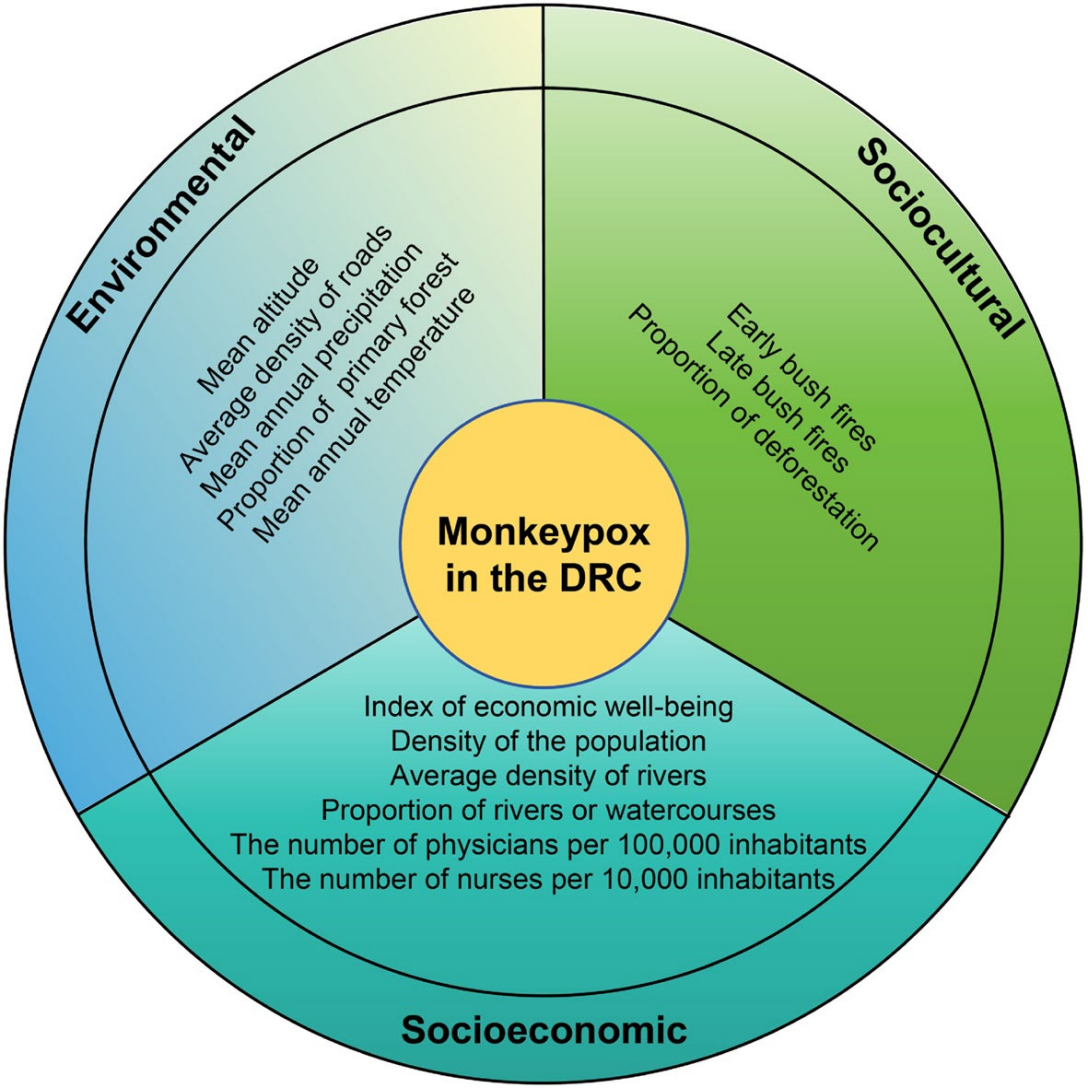
Environmental, socioeconomic, and sociocultural variables considered in this study, based on a One Health approach

A One Health concept [36] were used to consider the risk factors and to construct the risk analysis model (Fig. 2). The variable information, sources and definitions included in the analysis are shown in Table 1. The epidemiological data, along with environmental, socioeconomic, and sociocultural variables at the HZ level from 2000 to 2015, were utilized to predict the number of suspected mpox cases from 2016 to 2023. And the data on suspected mpox cases from 2016 to 2021 were instrumental in validating the consistency between our predicted values and the actual reported cases during that period.

**Table 1:** Variable information and definitions—Data layers, sources, and variables used in the prediction model

Layer/Variable	Proxy	Source	Variable definition
Epidemiological data			
Number of reported suspected mpox cases	Observed cases	DRC’s Ministry of Public Health (Direction of Disease Control)	Number of reported suspected mpox cases
Environmental variable			
Mean altitude	Altitude	http://srtm.csi.cgiar.org	Mean altitude of health zone
Average road density	DensRoads	https://data.humdata.org/dataset/dr-congo-settlements	Average road density in health zone
Mean annual precipitation	Precipitation	https://gmao.gsfc.nasa.gov/reanalysis/MERRA/	Mean annual precipitation in health zone
Proportion of primary forest	Primaryforest	ftp://congo.iluci.org/FACET/DRC/	Proportion of health zone area covered by primary forest
Mean annual temperature	Temperature	https://gmao.gsfc.nasa.gov/reanalysis/MERRA/	Mean annual temperature in health zone
Socioeconomic variable			
Index of economic well-being	IEW	Each district from the first two Demographic and Health Surveys (DHS) (Ministe’redu Plan, 2008, 2014)	Index of economic well-being as a proxy for socioeconomic status (poverty level)
Population density	PopDensity	http://esa.un.org/wpp/	Population of health zone divided by its surface area
Average river density	DensRivers	ftp://congo.iluci.org/FACET/DRC/	Average river density in health zone as a proxy for alternative feeding behavior (i.e., fish consumption)
Proportion of rivers or watercourses	PercentRivers	ftp://congo.iluci.org/FACET/DRC/	Proportion of health zone area intersected by rivers as a proxy for alternative feeding behavior (i.e., fish consumption)
Number of nurses per 10,000 inhabitants	NumNurses	https://www.caid.cd	Number of nurses per 10,000 inhabitants in each health zone
Number of physicians per 100,000 inhabitants	NumPhysicians	https://www.caid.cd	Number of physicians per 100,000 inhabitants in each health zone
Sociocultural variable			
Early bush fires	EarlyBfire	https://earthdata.nasa.gov/earth-observation-data/near-real-time/firms/active-fire-data	Early bush fires or fires that occurred in the first half of the year as a proxy for agricultural activities
Late bush fires	LateBfire	https://earthdata.nasa.gov/earth-observation-data/near-real-time/firms/active-fire-data	Late bush fires or fires that occurred in the second half of the year as a proxy for agricultural activities
Proportion of deforestation	Deforestation	ftp://congo.iluci.org/FACET/DRC/	Proportion of health zone area affected by deforestation. The deforestation variable was used as a proxy for high-risk human activities (agriculture, hunting, logging, charcoal production, and so on)

### Correlation analysis among environmental, socioeconomic, and sociocultural factors and mpox transmission

#### Construction of a negative binomial (NB) regression model

Considering possible overdispersion of the data, the NB regression model was used to determine the effect of environmental, socioeconomic, and sociocultural factors on human mpox transmission using software R 4.4.1 (TUNA Team, Tsinghua University, Beijing, China). The Poisson-gamma mixed distribution is the foundation of an NB regression model, which is useful in predicting count-based data [38]. Due to the non-negative integer values and the higher variance of suspected mpox cases, a variance inflation analysis was adopted to minimize multicollinearity. Variables with a variance inflation factor greater than 10 were excluded at this stage [39]. The regression coefficients were exponentiated to represent incidence rate ratios (IRRs) with their 95% confidence intervals (*CI*s).

#### Construction of a least absolute shrinkage and selection operator (LASSO) regression model

LASSO regression has proven effective in identifying disease-related influential variables in scenarios characterized by high dimensionality and multicollinearity among variables [28]. Moreover, LASSO regression can minimize the prediction error of continuous dependent variables by imposing constraints on the model parameters that cause the regression coefficients of certain predictor variables to decrease to zero. It has been demonstrated that the LASSO method improves overall prediction accuracy by reducing the coefficients of some eigenvariables in the model to zero, thus facilitating the selection of important variables among the many eigenvariables [29]. Therefore, the LASSO regression model was applied to identify environmental, socioeconomic, and sociocultural risk factors related to the incidence of mpox in the DRC. Variables with non-zero regression coefficients were considered risk factors that were strongly correlated with the response variable. In contrast, predictor variables with regression coefficients approaching zero after the contraction process were excluded from the model. To address the issues of overfitting and multicollinearity during model extrapolation, tenfold cross-validation was employed, involving the centralization and standardization of included variables to optimize the lambda parameter [40].

#### Principal component analysis (PCA)

PCA was applied to identify the key environmental, socioeconomic, and sociocultural variables contributing to the incidence of mpox. The variables included in the analysis are shown in Table 1. PCA is a multivariate method based on an eigenvector; it is not appropriate for use with uncorrelated variables. PCA can reveal the internal data structure through analyzing loadings of variables [30, 41]. PCA groups linear combinations of correlated variables together into principal components, but the components themselves are derived such that they are uncorrelated with each other [30]. PCA was performed using IBM SPSS statistics 27.0 software (International Business Machines Corporation, Armonk, USA). Factors with eigenvalues of at least 1.0 were retained. Varimax rotation was used to produce interpretable factors [42]. Variables with an absolute value of the coefficient greater than 0.5 were considered to be influential within each component.

### Prediction for the epidemic trend of mpox

#### Construction of the grey prediction model GM (1, n)

The principle of the grey prediction model involves establishing a dynamic model for the original time-series dataset, then accumulating the original series to generate a new accumulated series, constructing a new model based on this, and finally simplifying the results to generate new forecast values [43]. Generally, the grey prediction model is written as GM (m, n), where “m” is the order, and “n” is the number of variables in the modeling equation.. GM (1, 1) is one of the basic models of grey prediction, and the GM (1, n) model has the characteristics of including the driving effects among each influencing factor. The GM (1, n) model is a special grey prediction model. It performs prediction by dividing the time-series data into several grey systems, where “1” is the order of the differential equation, and “n” is the number of variables.

The GM (1, n) model was applied to predict the epidemic trend of mpox in the DRC using Python 3.12.1 (the Python Software Foundation, Beaverton, USA). Epidemiological data, alongside environmental, socioeconomic, and sociocultural variables collected from 2000 to 2015 in the DRC, were input into the GM (1, n) model to predict the number of suspected mpox cases from 2016 to 2023. Additionally, the predicted number of suspected mpox cases from 2016 to 2023 in the DRC was compared with the actual reported data from 2016 to 2021. This comparison was made to evaluate the consistency between the predicted and actual values. Such a way of validation was expected to better show the performance of the model. The construction of the GM (1, n) model is described below.

With original time-series data,

$${\text{x}}_{i}^{(0)}=({\text{x}}_{i}^{(0)}(1),{\text{x}}_{i}^{(0)}(2),\cdots ,{\text{x}}_{i}^{(0)}(\text{n}){)}_{.}$$

The original sequence ${\text{x}}_{i}{ }^{(0)}$ is first- accumulating to form ${\text{x}}_{i}{ }^{(1)}$

$${x}_{i}^{(1)}\left(k\right)=\sum_{i=1}^{l} {x}_{i}^{(0)}\left(l\right)$$

$$i=\text{1,2},\cdots ,n;l=\text{1,2},\cdots ,{k}_{.}$$

The ${x}_{1}{ }^{(1)}$ adjacent mean sequence ${z}_{1}{ }^{(1)}$ is generated as follows:

$${\text{z}}_{1}^{(1)}(\text{k})=0.5\times \left({\text{x}}_{1}^{(1)}(\text{k})+{\text{x}}_{1}^{(1)}(\text{k}-1)\right)$$

The GM (1, n) model is constructed using the following one-order linear differential equation:

$${x}_{1}^{(0)}(k)+a{z}_{1}^{(1)}(k)=\sum_{i=2}^{N} {b}_{i}{x}_{i}^{(1)}(k)$$

In the above formula, a is the development coefficient, which represents the development trend of the original series; b (the driving coefficient) reflects the relationship of change between system data. The values of a and b were estimated using the least squares method; the data were re-reduced by first-order subtraction to obtain the predicted value of the sequence.

## Results

**Fig. 3:**
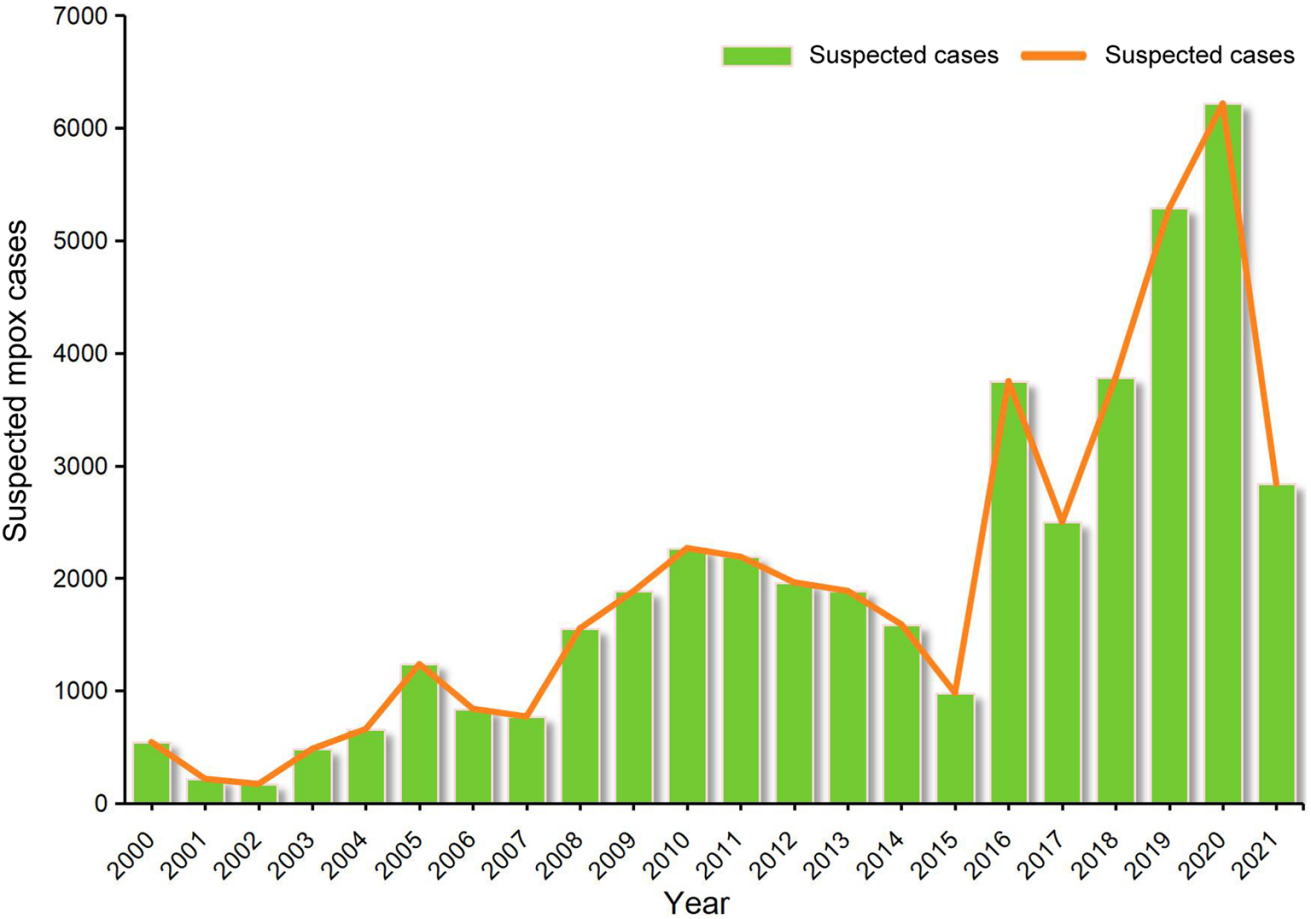
The trend of suspected human mpox cases reported in the Democratic Republic of the Congo from 2000 to 2021

Over the period 2000–2021, a total of 43,628 suspected mpox cases were reported in the DRC (Fig. 3). The number of suspected mpox cases reached the peak in 2020, with 6216 cases. The number of suspected cases from 2016 to 2021 accounted for over half the total number of cases reported over the study period in the DRC (24,379/43,628, 55.9%). Since 2016, the number of suspected mpox cases reported annually has been always higher than the number of cases in any individual year from 2000 to 2015.

### Risk factors associated with mpox transmission

#### NB regression model

The proportion of primary forest (IRR: 1.023, 95% *CI*: 1.018–1.027), index of economic well-being (IRR: 1.046, 95% *CI*: 1.039–1.052), and mean annual precipitation (IRR: 1.040, 95% *CI*: 1.031–1.049) were positively associated with the incidence of mpox in the NB regression model (Table 2). The IRR values can be interpreted as follows: a 1% increase in the area covered by primary forest in an HZ will result in a 2.3% increase in the mpox incidence rate; a 1% increase in the proportion of households in the lowest index of economic well-being quintile in an HZ will lead to a 4.6% increase in the mpox incidence rate; and a 1% increase in precipitation in an HZ will result in a 4.0% increase in the mpox incidence rate.

**Table 2:** Environmental, socioeconomic, and sociocultural variables associated with the incidence of mpox in the DRC from 2000 to 2015

Variable	IRR	2.5%	97.5%
Proportion of primary forest	1.023	1.018	1.027
Index of economic well-being	1.046	1.039	1.052
Mean annual precipitation	1.040	1.031	1.049

#### LASSO regression model

**Fig. 4:**
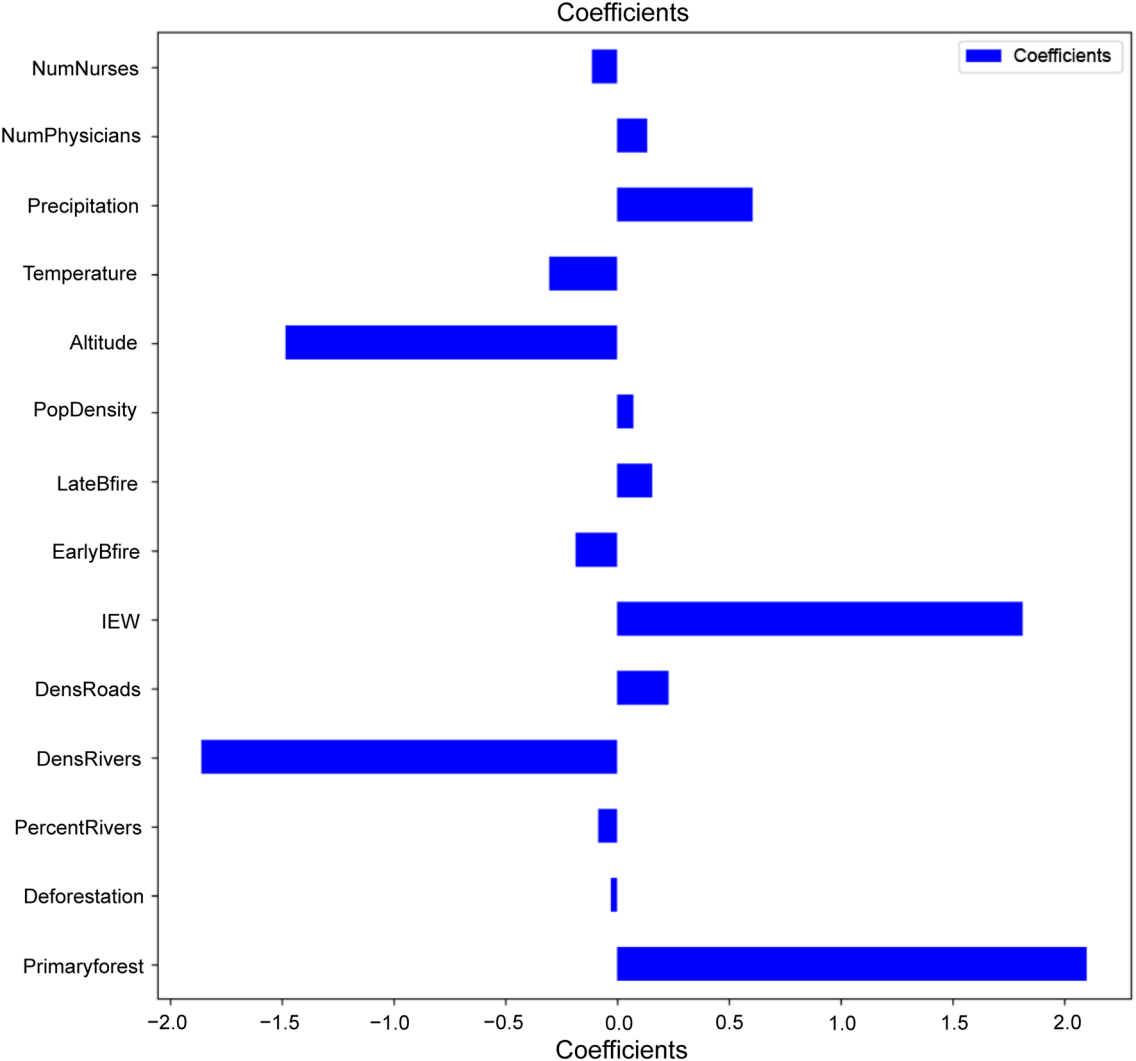
Model regression coefficient diagram of 14 environmental, socioeconomic, and sociocultural variables in the LASSO regression model

The LASSO regression algorithm in tenfold cross-validation identified that three factors had a significantly positive effect on the incidence of mpox, namely the proportion of primary forest, index of economic well-being, and mean annual precipitation (Fig. 4). The output of the LASSO regression model provided the following coefficients: 2.098, 1.811, 0.605. Moreover, two environmental variables were negatively correlated with the incidence of mpox, namely the mean altitude and average river density. The output of the LASSO regression model provided the following coefficients: − 1.857, − 1.482.

#### Principal component analysis

We identified five principal components within the environmental, socioeconomic, and sociocultural variables, explaining 69% of the variance in all variables (Table 3). Among these, the first principal component explained approximately 20% of the variance in all variables. The dominant variables in the first principal component were the number of nurses per 10,000 inhabitants, number of physicians per 100,000 inhabitants, index of economic well-being, and density of the population. The mean annual temperature, mean altitude, and average river density were the dominant variables for component 2 (18%). The mean annual precipitation, proportion of primary forest, and late bushfires were the dominant variables for component 3 (15%). The proportion of rivers or watercourses and average road density were the dominant variables for component 4 (9%), and early bushfires were the dominant variables for component 5 (7%).

**Table 3:** Five principal components obtained after varimax rotation within the environmental, socioeconomic, and sociocultural variables

Proxy of variables	Component				
	PC1	PC2	PC3	PC4	PC5
NumNurses	**0.885**^†^	0.102	− 0.019	0.137	0.036
PopDensity	**0.787**	0.039	− 0.028	− 0.064	0.004
NumPhysicians	**0.771**	0.094	− 0.043	0.208	0.029
IEW	− **0.586**	0.385	0.353	− 0.005	0.099
Temperature	0.193	**0.928**	0.095	0.057	− 0.002
Altitude	− 0.205	− **0.904**	− 0.166	− 0.115	− 0.022
DensRivers	− 0.307	**0.655**	0.117	− 0.252	− 0.126
Precipitation	− 0.081	0.200	**0.838**	− 0.112	− 0.011
Primaryforest	− 0.215	0.226	**0.807**	0.111	0.127
LateBfire	− 0.262	0.150	− **0.596**	0.029	0.247
PercentRivers	0.003	− 0.085	− 0.217	**0.789**	0.007
DensRoads	− 0.240	− 0.094	− 0.271	− **0.626**	0.046
EarlyBfire	− 0.099	0.096	0.123	0.120	− **0.842**
Proportion of variance (%)	19.76%	17.91%	14.88%	8.75%	7.27%
Cumulative variance (%)	19.76%	37.67%	52.55%	61.30%	68.57%*

*These components together explain 68.57% of the variance in environmental, socioeconomic, and sociocultural variables in the DRC

^†^Variables with an absolute value of at least 0.5 are shown in bold. A positive sign associated with a variable suggests that higher values of that variable have a significant influence on the component being studied

### Establishment and validation of the GM (1, n)-based prediction model for mpox incidence

**Fig. 5:**
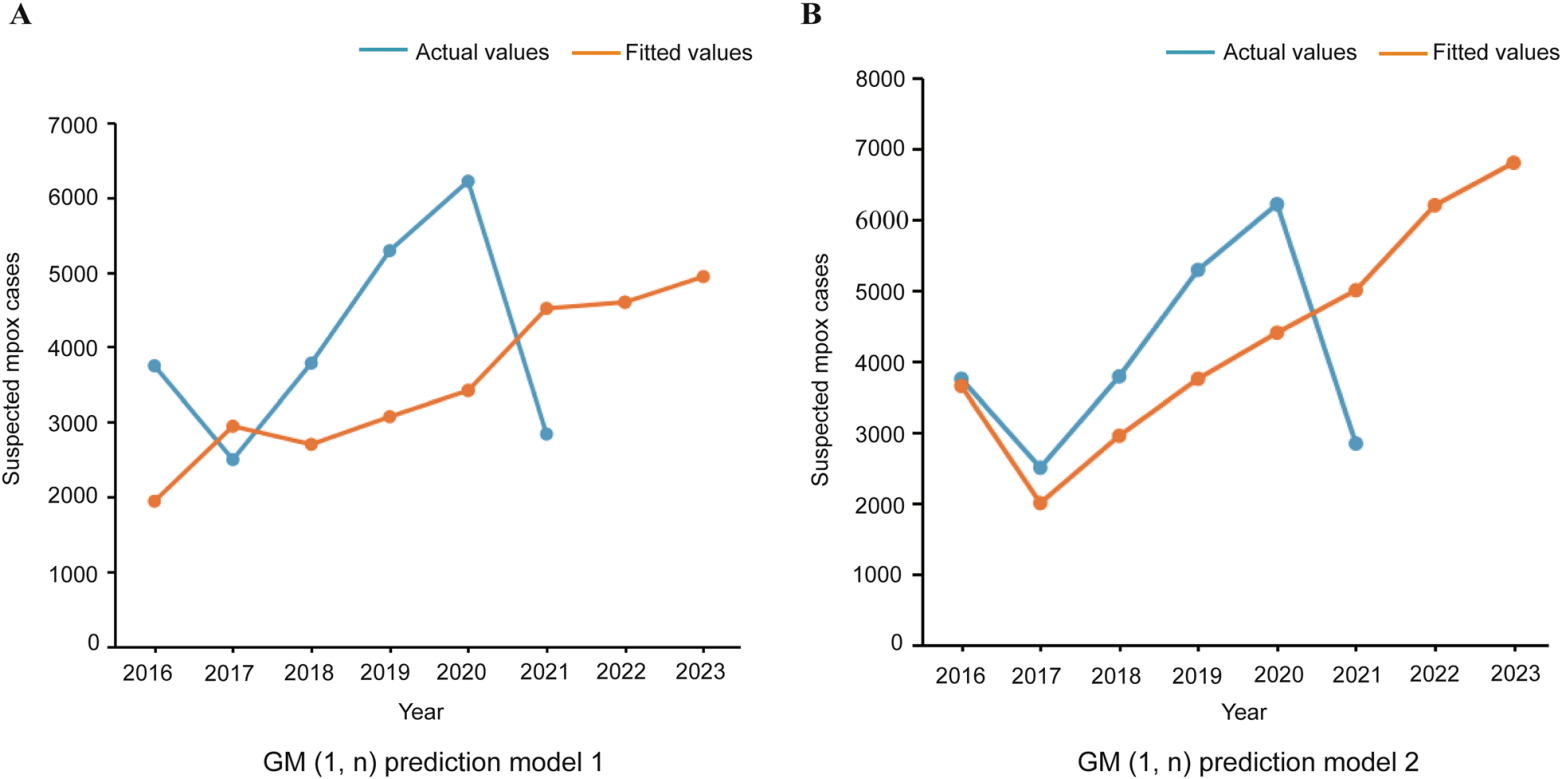
Results and fitting of the GM (1, n) prediction model. The horizontal axis shows the year, and the vertical axis shows the number of mpox cases. The predicted number of mpox cases in the DRC is shown in orange, and the actual value is shown in blue. **A** Fitting diagram of GM (1, n) prediction model 1 based on the reported number of suspected cases of mpox and all environmental, socioeconomic, and sociocultural variables. **B** Fitting diagram of GM (1, n) prediction model 2 based on the reported number of suspected mpox cases, proportion of primary forest, index of economic well-being, and mean annual precipitation

The reported number of suspected mpox cases and all environmental, socioeconomic, and sociocultural variables from 2000 to 2015 in the DRC were taken as an original time series to build the prediction model for mpox, viz., GM (1, n) prediction model 1 (Fig. 5A). Moreover, the reported number of suspected mpox cases, proportion of primary forest, index of economic well-being, and mean annual precipitation from 2000 to 2015 in the DRC were used as an original time series to build GM (1, n) prediction model 2 (Fig. 5B).

Extrapolated prediction was performed based on GM (1, n) prediction models 1 and 2 to estimate the incidence of mpox from 2016 to 2023 (Fig. 5A, B). A comparison of the actual reported number of suspected mpox cases from 2016 to 2021 with the results of GM (1, n) prediction models 1 and 2 showed that although there appeared to be a slight underestimation of mpox cases by GM (1, n) prediction model 1 from 2018 to 2020, GM (1, n) prediction model 2 (with the reported number of suspected mpox cases, proportion of primary forest, index of economic well-being, and mean annual precipitation) showed good performance in predicting the incidence of mpox. The relative error (RE) was used to evaluate the models’ performance. The RE of prediction models l and 2 was 2.71 and 2.69, respectively.

## Discussion

Understanding the risk factors of mpox transmission through the One Health concept offers an effective means for the prevention and control of the disease. Our study used the One Health concept [36] to re-categorized a total of fourteen variables into the environmental, socioeconomic, and sociocultural factors and considered their associations with the mpox transmission in the DRC. The NB and LASSO regression models showed that the incidence of mpox in the DRC was positively associated with the proportion of primary forest, index of economic well-being, and mean annual precipitation. The PCA identified five principal components that explained 69% of the variance in the environmental, socioeconomic, and sociocultural variables. Finally, the established GM (1, n) model, which was used to predict the epidemic trend of mpox, using the reported number of suspected mpox cases, proportion of primary forest, index of economic well-being, and mean annual precipitation, effectively predicted the epidemic trend.

Interestingly, our study demonstrated that socioeconomic factors had the most important role in mpox transmission. The economic well-being index, a measure of poverty [26], was positively associated with mpox incidence. Populations with low socioeconomic status have been largely demonstrated to be associated with the burdens of many infectious diseases [25, 44, 45]. In the DRC, many individuals consume bushmeat owing to poverty [25], which increases the risk of zoonotic diseases like mpox. However, most of the population in the DRC resides in rural areas surrounded by forests, so the patterns of human behavior and social activities primarily rely on forest resources to fulfill individuals' material and nutritional needs, which increases the risk of spillover of human and animal diseases to some extent [25]. Therefore, reducing reliance on wildlife as a food source by addressing food security in the DRC could decrease human contact with potentially infected animals, thereby reducing the risks for many zoonotic diseases [46]. Efforts could be made to provide health education regarding the risk of zoonotic disease spillover, train community health workers, and ensure proper delivery of safe medicines and commodities to those with low economic status [47, 48].

Our study also highlighted the role of environmental factors in predicting the incidence of mpox. Changes in primary forest cover and human–nature interactions in the DRC could increase contact with MPXV reservoirs, elevating the transmission risk. For example, increased primary forest cover can interact with human land-use activities in intricate ways, influenced by various socioeconomic and policy contexts [49]. For example, enhanced forest cover can promote biodiversity and provide vital ecosystem services that support local livelihoods; however, this may also lead to conflicts over land use, particularly in areas prioritizing agricultural expansion or urban development [50]. In contrast, ecological disturbances resulting from the geographic expansion of human land-use activities, such as deforestation, may enhance human exposure to rodents and other suspected reservoir species found in primary forests [51]. This increased interaction can elevate the risk of zoonotic spillover and the emergence of infectious diseases, including mpox [19]. Understanding these dynamics is vital for addressing the drivers of mpox transmission and developing integrated strategies that align human health with environmental sustainability within a One Health framework. Effective policy frameworks that incentivize sustainable land use and conservation are essential for mitigating these conflicts and promoting harmony between ecological preservation and economic growth.

Different from the findings of Mandja et al. (2022), precipitation was identified as a major predictor of mpox transmission in the DRC. Over the past two decades, annual precipitation has increased in most parts of the African continent, including the southeastern part of the DRC [52, 53]. Such changes in climate variables could have an effect on the transmission or distribution of infectious diseases, particularly zoonotic infectious diseases [54–57]. For example, mpox outbreaks usually occur during the fall as a result of increased rainfall that causes flooding and deforestation; both of these factors drive animals (i.e., potential reservoir hosts) into human populations and residential areas [58]. However, such a correlation between annual precipitation and human mpox occurrence is not always consistent [20]. These findings further emphasize the importance of understanding the interconnection between humans, plants, animals, and their shared environment in the prevention and control of mpox [24, 46].

Choosing the optimal model highly depends on the type of data available. In our study, we initially used a Poisson regression model to analyze risk factors associated with mpox transmission. However, our analysis revealed that the dependent variable exhibited over-dispersion. Consequently, we opted for a negative binomial (NB) regression model to better account for this characteristic of the data. In the analysis of the prediction for the epidemic trend of mpox, different from the methods of previous studies using the time-series model to predict the epidemic trend of mpox [43], the GM (1, n) prediction model established in this study was based on the proportion of primary forest, index of economic well-being, and mean annual precipitation to predict the epidemic trend of mpox and validated using data from 2016 to 2021 in the DRC. Compared with traditional time series forecasting models, the modeling process of multivariable GM (1, n) model takes full account of the effect of the relevant factors on the system change, ensuring accurate and reliable prediction [31, 32]. The developed model exhibited an optimal ability to make well-fitting predictions. The model indicated that considering the risk of mpox, the development of interventions to prevent and control mpox at the human–animal–ecosystem interface is crucial [46]. For example, increasing awareness in local communities regarding the risks of deforestation and mpox transmission could be achieved through education programs and forest conservation measures [59]. Considering multidisciplinary and multisectoral cooperation linking public health agencies, agricultural, animal health sectors, and environmental health and conservation sectors may offer the best path to promote the implementation of such programs. The predicted values from the GM (1, n) model indicated an upward trend from 2016 to 2023. In contrast, the actual number of suspected mpox cases reported between 2020 and 2021 exhibited a downward trend. This discrepancy may be attributed to the COVID-19 pandemic, which overwhelmed health systems in Africa [60], thereby hindering the detection capacity of the surveillance system in the DRC. Consequently, this situation led to a decline in the reporting of other diseases, including mpox. Future research should consider to use various artificial intelligence and machine learning techniques (e.g., Stacking Ensemble Learning, NeuralProphet, Hybrid Convolutional Neural Network-Long Short Term Memory model) to forecast the mpox transmission in the DRC [61].

This study has several limitations. First, this study used suspected cases over confirmed mpox cases as confirmation of MPXV infection for many cases was always not possible in DRC [35, 62]. Second, we did not consider certain variables that may change over time, such as viral mutations, which were critical factors in the mpox pandemic from 2022 to 2024 [12, 63]. Similarly, we assumed that the capacity of surveillance in the DRC did not change over the past two decades. Furthermore, although we incorporated multiple factors at the environmental, socioeconomic, and sociocultural levels to understand the risk factors for mpox transmission, some factors that could influence the number of mpox cases were not included owing to the limitation of data availability, such as the history of vaccination against smallpox, sexual history, HIV status, and the history of close physical contacts with mpox cases [64, 65]. Moreover, although we consider using the One Health concept to consider risk factors for mpox transmission, indicators on animal were not included according to this concept. In addition, the considered variables in the proposed domains (e.g., environmental, socioeconomic, and sociocultural variables) may not comprehensive. For example, we may not include the household size, household income, consumption of wildlife in the domain of socioeconomic factors. Future research could consider to these variables under the One Health concept, while, it is often challenged by the effective collaboration across disciplines and sectors. In coping with this, the WHO is a member of the One Health Quadripartite with the Food and Agriculture Organization, the World Organisation for Animal Health and the United Nations Environment Programme, which plays a central role in promoting and coordinating a global One Health approach [66, 67].

## Conclusions

The incidence of mpox was found to be associated with the proportion of primary forest, index of economic well-being, and mean annual precipitation in the DRC. The GM (1, n) prediction model showed good performance in predicting the epidemic trend of mpox in the DRC. Our study further highlighted that the health of humans, animals, and the environment are linked and must be treated as a whole to explain and address the transmission and emergence of mpox outbreaks in the DRC, in line with the concept of One Health.

## Data Availability

Data sources and links have been explained in the manuscript.

## Abbreviations

Mpox: Monkeypox
MPXV: Monkeypox virus
DRC: Democratic Republic of the Congo
PHEIC: Public health emergency of international concern
HZ: Health zone
GLMM: Generalized linear mixed model
LASSO: Least absolute shrinkage and selection operator
NB: Negative binomial
IRRs: Incidence rate ratios
*CI*s: Confidence intervals
PCA: Principal component analysis
GM: Grey model
RE: Relative error
